# Di­aqua­bis­[*N*-(2-fluoro­benz­yl)-*N*-nitroso­hydroxy­laminato-κ^2^ *O*,*O*′]nickel(II)

**DOI:** 10.1107/S1600536814002876

**Published:** 2014-02-15

**Authors:** Olga Kovalchukova, Ali Sheikh Bostanabad, Adam Stash, Svetlana Strashnova, Igor Zyuzin

**Affiliations:** a Peoples’ Friendship University of Russia, 6 Miklukho-Mallaya, 117198 Moscow, Russia; b Karpov Institute of Physical Chemistry, 10 Vorontsovo Pole, 105064 Moscow, Russia; c The Institute of Problems of Chemical Physics of the Russian Academy of Sciences (IPCP RAS), Academician Semenov Avenue 1, Chernogolovka, Moscow Region, 142432 , Russian Federation

## Abstract

In the centrosymmetric title compound, [Ni(C_7_H_6_FN_2_O_2_)_2_(H_2_O)_2_], the Ni^II^ cation is in a slightly distorted octa­hedral environment and is surrounded by four O atoms from the N—O groups of the organic ligands [Ni—O = 2.0179 (13) and 2.0283 (12) Å], and two water mol­ecules [Ni—O = 2.0967 (14) Å]. The *N*-(2-fluoro­benz­yl)-*N*-nitroso­hydroxy­laminate monoanions act as bidentate chelating ligands. In the crystal, the Ni cations in the columns are shifted in such a way that the coordinated water mol­ecules are involved in the formation of hydrogen bonds with the O atoms of the organic species of neighbouring mol­ecules. Thus, a two-dimensional network parallel to (100) is built up by hydrogen-bonded molecules.

## Related literature   

For the synthesis of the potassium *N*-(2-fluoro­benz­yl)-*N*-nitroso­hydroxy­laminate salt, see: Zyuzin *et al.* (1997 ▶) and of the Ni complex of *N*-(2-fluoro­benz­yl)-*N*-nitroso­hydroxy­laminate, see: Kovalchukova *et al.* (2013 ▶). For the structures of some 3d-metal complexes with *N*-nitroso­hydroxyl­amine deriv­atives, see: Deák *et al.* (1998 ▶); Okabe & Tamaki (1995 ▶); Tamaki & Okabe (1996 ▶, 1998 ▶). For the synthesis, properties and applications of other metal nitroso­hydroxy­laminates, see: Okabe *et al.* (1995 ▶); Abraham *et al.* (1987 ▶); Venter *et al.* (2009 ▶); Popov & Wendlandt (1954 ▶); Lundell & Knowles (1920 ▶); Buscarons & Canela (1974 ▶); Oztekin & Erim (2000 ▶); Yi *et al.* (1995 ▶); McGill *et al.* (2000 ▶); Shiino *et al.* (2001 ▶). 
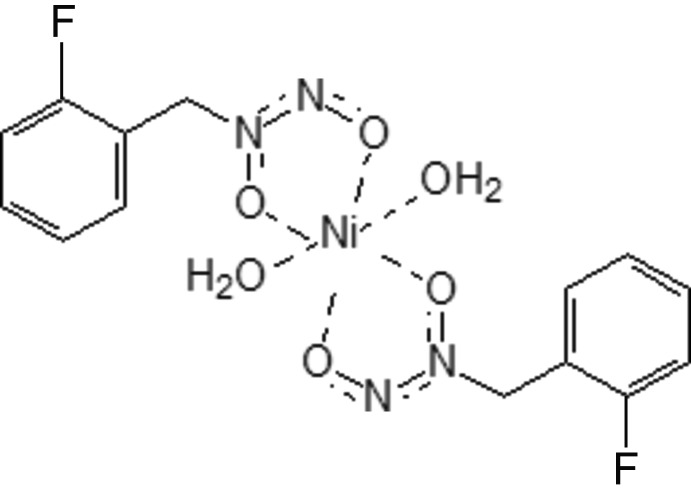


## Experimental   

### 

#### Crystal data  

[Ni(C_7_H_6_FN_2_O_2_)_2_(H_2_O)_2_]
*M* *_r_* = 433.02
Monoclinic, 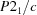

*a* = 15.411 (3) Å
*b* = 7.235 (1) Å
*c* = 7.604 (1) Å
β = 91.65 (3)°
*V* = 847.5 (3) Å^3^
*Z* = 2
Mo *K*α radiation
μ = 1.21 mm^−1^
*T* = 293 K
0.75 × 0.20 × 0.05 mm

#### Data collection  

Enraf–Nonius CAD-4 diffractometer
Absorption correction: part of the refinement model (Δ*F*) (Walker & Stuart, 1983 ▶) *T*_min_ = 0.427, *T*_max_ = 0.809
1703 measured reflections
1571 independent reflections
1181 reflections with *I* > 2σ(*I*)
*R*_int_ = 0.022
3 standard reflections every 60 min intensity decay: 0.0%

#### Refinement  

*R*[*F* ^2^ > 2σ(*F* ^2^)] = 0.022
*wR*(*F* ^2^) = 0.066
*S* = 1.01
1571 reflections
132 parameters
2 restraints
H atoms treated by a mixture of independent and constrained refinement
Δρ_max_ = 0.31 e Å^−3^
Δρ_min_ = −0.32 e Å^−3^

### 

Data collection: *CAD-4-PC* (Enraf–Nonius, 1993 ▶); cell refinement: *CAD-4-PC*; data reduction: *CAD-4-PC*; program(s) used to solve structure: *SHELXS97* (Sheldrick, 2008 ▶); program(s) used to refine structure: *SHELXL97* (Sheldrick, 2008 ▶); molecular graphics: *SHELXTL* (Sheldrick, 2008 ▶); software used to prepare material for publication: *CIFTAB97* (Sheldrick, 2008 ▶) and *SHELX97*.

## Supplementary Material

Crystal structure: contains datablock(s) I, global. DOI: 10.1107/S1600536814002876/bv2230sup1.cif

Structure factors: contains datablock(s) I. DOI: 10.1107/S1600536814002876/bv2230Isup2.hkl

CCDC reference: 985943

Additional supporting information:  crystallographic information; 3D view; checkCIF report

## Figures and Tables

**Table 1:** Hydrogen-bond geometry (Å, °)

*D*—H⋯*A*	*D*—H	H⋯*A*	*D*⋯*A*	*D*—H⋯*A*
O3—H31⋯O1^i^	0.84 (1)	1.97 (1)	2.7987 (18)	169 (3)
O3—H32⋯O2^ii^	0.84 (1)	1.98 (1)	2.8078 (18)	170 (2)

Symmetry codes: (i) 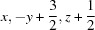; (ii) 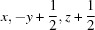.

**Fig. 1.**
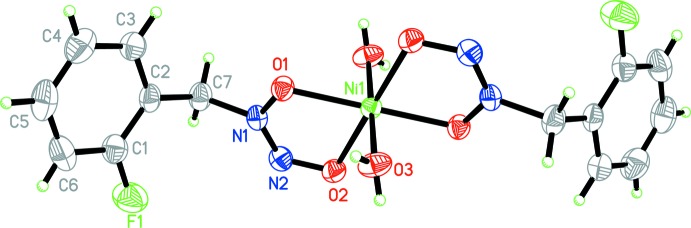
ORTEP view of C14H16F2N4O6 with atom labeling scheme (displacement ellipsoids are drawn at the 50% probability level for non-hydrogen atoms).

**Fig. 2.**
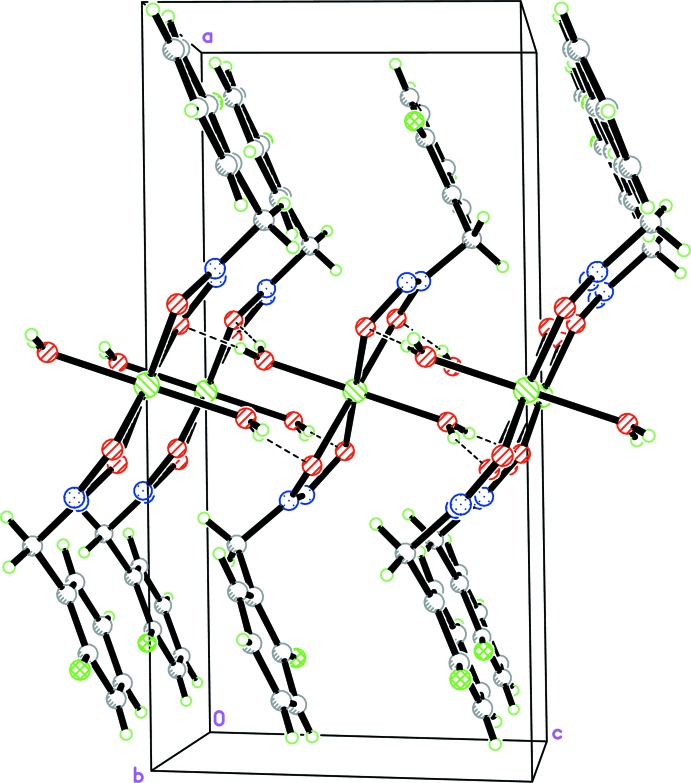
Molecular packing in the crystal of the complex along the crystallographic axis b.

**Fig. 3.**
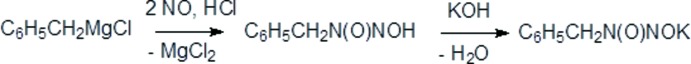
The synthesis of the potassium N-(2-fluorobenzyl)-Nnitrosohydroxylaminate salt

## supplementary crystallographic information

### 1. Comment

*N*-nitrosohydroxylamine derivatives are good chelating agents which form stable complexes with a wide range of metal ions (Okabe *et al.*, 1995; Abraham *et al.*, 1987; Venter *et al.*, 2009; Popov & Wendlandt, 1954). The phenyl and naphthyl derivatives, known as cupferron and neocupferron, are reported as good analytical reagents for the determination of zirconium in its ores and metallurgical products, as well as for the separation of iron and titanium from manganese and aluminum in limestone analysis, separation and direct UV detection of lanthanides and other analyses (Lundell & Knowles, 1920; Buscarons & Canela, 1974; Oztekin & Erim, 2000). *R*_2_N[N_2_0_2_] anions are smooth nonenzymatic releasers of nitric oxide in physiological media (Yi *et al.*, 1995; McGill *et al.*, 2000) and possess the property of inhibition of mushroom tyrosinase (Shiino *et al.*, 2001).

In the title compound, C_14_H_16_F_2_N_4_NiO_6_, the Ni cation of the centrosymmetrical structure is in a slightly distorted octahedral coordination and is surrounded by four oxo O atoms of the N—O groups of the organic ligands [Ni—O = 2.0179 (13) and 2.0283 (12) Å], and two water molecules in the axial positions [Ni—O = 2.0967 (14) Å]. The described coordination type of the central atom correlates with those described previously for the dimethanolo-bis(N– nitroso-*N*-phenyl-hydroxylaminato-o,o) cobalt(II) (Deak *et al.*, 1998) and the dimethanolo-bis (*N*-nitroso-*N*-phenylhydroxylaminato-O,*O*')nickel(II) (Okabe & Tamaki, 1995). On the other hand, in the reported structure of the diaquabis[*N*-(1-naphthyl)-*N*-nitrosohydroxylaminato- O, O'] cobalt(II) (Tamaki & Okabe, 1998), the two coordinated water molecules are in the *cis* arrangement. In addition in the bis(*N*-nitroso-*N*-phenylhydroxylaminato)manganese, or manganese cupferronate (Tamaki & Okabe, 1996), the saturation of the coordination sphere of the Mn(II) cation occurs with the two O atoms of the nitroso groups of two adjacent cupferron ligands. The *N*-(2-fluorobenzyl)-*N*-nitrosohydroxylaminate monoanions act as bidentate chelating ligands. The Ni cations in the columns are shifted in such a way that the coordinated H_2_O molecules are involved in the formation of hydrogen bonds with the O atoms of the organic species of the neighbouring molecules in the columns. Thus, the crystal lattice of the reported structure is a supramolecular architecture built up by infinite one dimensional chains of hydrogen bonded molecules.

### 2. Experimental

The potassium *N*-(2-fluorobenzyl)-*N*-nitrosohydroxylaminate salt and its Ni complex was prepared (see Fig. 3) according to procedures described previously (Zyuzin *et al.*, 1997; Kovalchukova *et al.*, 2013). Single crystals of C_14_H_16_F_2_N_4_O_6_ were grown by the slow evaporation of the ethanol solution of the diaquabis[N-(2-fluorobenzyl)-*N*-nitrosohydroxylaminato-*O*,*O*']nickel(II) powdered sample.

### 3. Refinement

The structure of of C_14_H_16_F_2_N_4_O_6_ was solved by direct method and all non-hydrogen atoms were located and refined in anisotropically. All the hydrogen atoms were located in difference electron density syntheses and included in refinement with fixed parameters.

### Crystal data

**Table:**

[Ni(C_7_H_6_FN_2_O_2_)_2_(H_2_O)_2_]	*F*(000) = 444
*M**_r_* = 433.02	*D*_x_ = 1.697 Mg m^−^^3^
Monoclinic, *P*2_1_/*c*	Mo *K*α radiation, λ = 0.71073 Å
*a* = 15.411 (3) Å	Cell parameters from 24 reflections
*b* = 7.235 (1) Å	θ = 10.9–12.5°
*c* = 7.604 (1) Å	µ = 1.21 mm^−^^1^
β = 91.65 (3)°	*T* = 293 K
*V* = 847.5 (3) Å^3^	Plate, green
*Z* = 2	0.75 × 0.20 × 0.05 mm

### Data collection

**Table:**

Enraf–Nonius CAD-4 diffractometer	1181 reflections with *I* > 2σ(*I*)
Radiation source: fine-focus tube	*R*_int_ = 0.022
β-filter monochromator	θ_max_ = 25.5°, θ_min_ = 2.6°
ω/2θ scans	*h* = −18→18
Absorption correction: part of the refinement model (Δ*F*) (Walker & Stuart, 1983)	*k* = 0→8
*T*_min_ = 0.427, *T*_max_ = 0.809	*l* = 0→9
1703 measured reflections	3 standard reflections every 60 min
1571 independent reflections	intensity decay: 0.0%

### Refinement

**Table:**

Refinement on *F*^2^	Primary atom site location: structure-invariant direct methods
Least-squares matrix: full	Secondary atom site location: difference Fourier map
*R*[*F*^2^ > 2σ(*F*^2^)] = 0.022	Hydrogen site location: difference Fourier map
*wR*(*F*^2^) = 0.066	H atoms treated by a mixture of independent and constrained refinement
*S* = 1.01	*w* = 1/[σ^2^(*F*_o_^2^) + (0.0471*P*)^2^] where *P* = (*F*_o_^2^ + 2*F*_c_^2^)/3
1571 reflections	(Δ/σ)_max_ < 0.001
132 parameters	Δρ_max_ = 0.31 e Å^−^^3^
2 restraints	Δρ_min_ = −0.32 e Å^−^^3^

### Special details

**Table:**

Geometry. All e.s.d.'s (except the e.s.d. in the dihedral angle between two l.s. planes) are estimated using the full covariance matrix. The cell e.s.d.'s are taken into account individually in the estimation of e.s.d.'s in distances, angles and torsion angles; correlations between e.s.d.'s in cell parameters are only used when they are defined by crystal symmetry. An approximate (isotropic) treatment of cell e.s.d.'s is used for estimating e.s.d.'s involving l.s. planes.
Refinement. Refinement of *F*^2^ against ALL reflections. The weighted *R*-factor *wR* and goodness of fit *S* are based on *F*^2^, conventional *R*-factors *R* are based on *F*, with *F* set to zero for negative *F*^2^. The threshold expression of *F*^2^ > σ(*F*^2^) is used only for calculating *R*-factors(gt) *etc*. and is not relevant to the choice of reflections for refinement. *R*-factors based on *F*^2^ are statistically about twice as large as those based on *F*, and *R*- factors based on ALL data will be even larger.

### Fractional atomic coordinates and isotropic or equivalent isotropic displacement parameters (Å^2^)

**Table:**

	*x*	*y*	*z*	*U*_iso_*/*U*_eq_	
Ni1	0.5000	0.5000	0.5000	0.02414 (12)	
F1	0.12983 (10)	0.46369 (18)	0.3199 (2)	0.0610 (4)	
O1	0.39701 (8)	0.63578 (16)	0.39297 (15)	0.0292 (3)	
O2	0.41642 (8)	0.28946 (16)	0.44659 (16)	0.0312 (3)	
N1	0.34766 (9)	0.51068 (19)	0.31185 (19)	0.0278 (3)	
N2	0.35419 (10)	0.3370 (2)	0.3362 (2)	0.0317 (3)	
C1	0.14337 (13)	0.6479 (3)	0.3049 (3)	0.0386 (4)	
C2	0.22021 (12)	0.7072 (3)	0.2360 (2)	0.0329 (4)	
C3	0.23158 (14)	0.8967 (3)	0.2205 (3)	0.0406 (5)	
H3	0.2824	0.9423	0.1738	0.049*	
C4	0.16858 (17)	1.0184 (3)	0.2733 (3)	0.0519 (6)	
H4	0.1773	1.1451	0.2629	0.062*	
C5	0.09283 (16)	0.9527 (3)	0.3412 (3)	0.0531 (6)	
H5	0.0504	1.0354	0.3760	0.064*	
C6	0.07923 (13)	0.7649 (3)	0.3581 (3)	0.0477 (5)	
H6	0.0282	0.7193	0.4041	0.057*	
C7	0.28684 (13)	0.5742 (3)	0.1721 (2)	0.0369 (4)	
H71	0.2575	0.4679	0.1206	0.044*	
H72	0.3194	0.6333	0.0805	0.044*	
O3	0.45896 (10)	0.53062 (17)	0.75889 (18)	0.0371 (3)	
H31	0.4344 (17)	0.628 (3)	0.790 (4)	0.075 (9)*	
H32	0.4418 (15)	0.443 (2)	0.821 (3)	0.052 (7)*	

### Atomic displacement parameters (Å^2^)

**Table:**

	*U*^11^	*U*^22^	*U*^33^	*U*^12^	*U*^13^	*U*^23^
Ni1	0.02426 (18)	0.02405 (17)	0.02409 (17)	0.00166 (12)	0.00050 (11)	0.00039 (11)
F1	0.0577 (8)	0.0447 (7)	0.0812 (10)	−0.0079 (6)	0.0135 (7)	−0.0001 (6)
O1	0.0292 (6)	0.0261 (5)	0.0321 (6)	0.0014 (5)	−0.0027 (5)	−0.0010 (5)
O2	0.0330 (7)	0.0271 (6)	0.0335 (6)	−0.0003 (5)	−0.0004 (5)	0.0022 (5)
N1	0.0243 (7)	0.0317 (7)	0.0274 (7)	0.0030 (6)	−0.0003 (6)	−0.0027 (6)
N2	0.0304 (8)	0.0317 (8)	0.0331 (7)	0.0006 (6)	0.0021 (6)	−0.0030 (6)
C1	0.0366 (11)	0.0405 (10)	0.0382 (10)	0.0008 (8)	−0.0049 (8)	−0.0023 (8)
C2	0.0286 (9)	0.0419 (10)	0.0279 (9)	0.0051 (7)	−0.0059 (7)	−0.0005 (7)
C3	0.0381 (11)	0.0441 (11)	0.0394 (10)	0.0001 (8)	−0.0046 (9)	0.0047 (8)
C4	0.0595 (14)	0.0403 (11)	0.0553 (13)	0.0083 (10)	−0.0067 (11)	0.0006 (9)
C5	0.0494 (13)	0.0590 (14)	0.0504 (13)	0.0228 (11)	−0.0050 (10)	−0.0083 (10)
C6	0.0317 (11)	0.0679 (15)	0.0434 (11)	0.0052 (10)	0.0013 (9)	−0.0062 (10)
C7	0.0351 (10)	0.0484 (10)	0.0269 (9)	0.0077 (9)	−0.0029 (8)	−0.0001 (8)
O3	0.0518 (8)	0.0300 (7)	0.0303 (6)	0.0022 (6)	0.0127 (6)	0.0008 (5)

### Geometric parameters (Å, º)

**Table:**

Ni1—O1^i^	2.0179 (13)	C2—C3	1.388 (3)
Ni1—O1	2.0179 (13)	C2—C7	1.498 (3)
Ni1—O2	2.0283 (12)	C3—C4	1.379 (3)
Ni1—O2^i^	2.0283 (12)	C3—H3	0.9300
Ni1—O3	2.0967 (14)	C4—C5	1.375 (4)
Ni1—O3^i^	2.0967 (14)	C4—H4	0.9300
F1—C1	1.354 (2)	C5—C6	1.381 (3)
O1—N1	1.3233 (19)	C5—H5	0.9300
O2—N2	1.302 (2)	C6—H6	0.9300
N1—N2	1.274 (2)	C7—H71	0.9700
N1—C7	1.470 (2)	C7—H72	0.9700
C1—C6	1.371 (3)	O3—H31	0.838 (10)
C1—C2	1.377 (3)	O3—H32	0.837 (10)
			
O1^i^—Ni1—O1	180.0	C1—C2—C3	116.90 (18)
O1^i^—Ni1—O2	101.70 (5)	C1—C2—C7	121.86 (17)
O1—Ni1—O2	78.30 (5)	C3—C2—C7	121.17 (18)
O1^i^—Ni1—O2^i^	78.30 (5)	C4—C3—C2	121.0 (2)
O1—Ni1—O2^i^	101.70 (5)	C4—C3—H3	119.5
O2—Ni1—O2^i^	180.0	C2—C3—H3	119.5
O1^i^—Ni1—O3	85.86 (6)	C5—C4—C3	120.1 (2)
O1—Ni1—O3	94.14 (6)	C5—C4—H4	120.0
O2—Ni1—O3	93.46 (6)	C3—C4—H4	120.0
O2^i^—Ni1—O3	86.54 (6)	C4—C5—C6	120.5 (2)
O1^i^—Ni1—O3^i^	94.14 (6)	C4—C5—H5	119.7
O1—Ni1—O3^i^	85.86 (6)	C6—C5—H5	119.7
O2—Ni1—O3^i^	86.54 (6)	C1—C6—C5	117.8 (2)
O2^i^—Ni1—O3^i^	93.46 (6)	C1—C6—H6	121.1
O3—Ni1—O3^i^	180.0	C5—C6—H6	121.1
N1—O1—Ni1	106.85 (9)	N1—C7—C2	113.27 (14)
N2—O2—Ni1	112.47 (10)	N1—C7—H71	108.9
N2—N1—O1	124.40 (14)	C2—C7—H71	108.9
N2—N1—C7	117.37 (14)	N1—C7—H72	108.9
O1—N1—C7	117.96 (14)	C2—C7—H72	108.9
N1—N2—O2	114.08 (14)	H71—C7—H72	107.7
F1—C1—C6	117.94 (19)	Ni1—O3—H31	120 (2)
F1—C1—C2	118.37 (17)	Ni1—O3—H32	124.0 (18)
C6—C1—C2	123.69 (19)	H31—O3—H32	109 (3)

Symmetry code: (i) −*x*+1, −*y*+1, −*z*+1.

### Hydrogen-bond geometry (Å, º)

**Table:**

*D*—H···*A*	*D*—H	H···*A*	*D*···*A*	*D*—H···*A*
O3—H31···O1^ii^	0.84 (1)	1.97 (1)	2.7987 (18)	169 (3)
O3—H32···O2^iii^	0.84 (1)	1.98 (1)	2.8078 (18)	170 (2)

Symmetry codes: (ii) *x*, −*y*+3/2, *z*+1/2; (iii) *x*, −*y*+1/2, *z*+1/2.
